# Supplementary material for: Single-cell multi-omics analysis of human testicular germ cell tumor reveals its molecular features and microenvironment
Source: Nat Commun. 2023 Dec 20;14:8462. doi: 10.1038/s41467-023-44305-9 (PMC10733385; doi:10.1038/s41467-023-44305-9)
Supplement: Supplementary file 1 — Supplementary Information [file 41467_2023_44305_MOESM1_ESM.pdf]

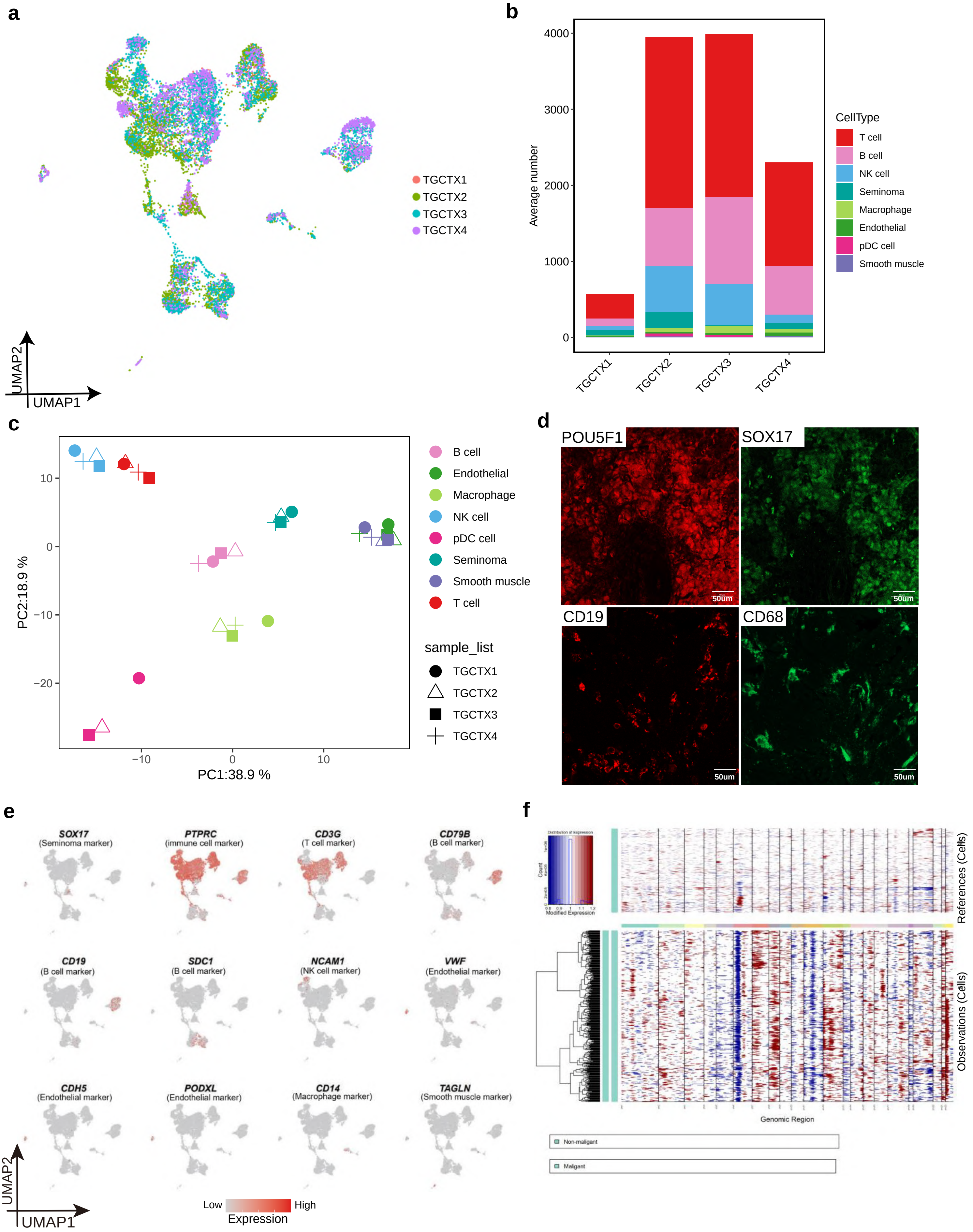
a
TGCTX1
TGCTX2
TGCTX3
TGCTX4
UMAP2
UMAP1
b
Average number
4000
3000
2000
1000
0
TGCTX1
TGCTX2
TGCTX3
TGCTX4
CellType
T cell
B cell
NK cell
Seminoma
Macrophage
Endothelial
pDC cell
Smooth muscle
c
PC2:18.9 %
PC1:38.9 %
10
0
−10
−20
B cell
Endothelial
Macrophage
NK cell
pDC cell
Seminoma
Smooth muscle
T cell
sample_list
TGCTX1
TGCTX2
TGCTX3
TGCTX4
d
POU5F1
SOX17
CD19
CD68
50um
e
SOX17
(Seminoma marker)
PTPRC
(immune cell marker)
CD3G
(T cell marker)
CD79B
(B cell marker)
CD19
(B cell marker)
SDC1
(B cell marker)
NCAM1
(NK cell marker)
VWF
(Endothelial marker)
CDH5
(Endothelial marker)
PODXL
(Endothelial marker)
CD14
(Macrophage marker)
TAGLN
(Smooth muscle marker)
UMAP2
UMAP1
Low
High
Expression
f
Distribution of Expression
Count
Modified Expression
References (Cells)
Observations (Cells)
Genomic Region
Non-maligant
Maligant

Supplementary Figure 1. Single-cell transcriptome profiling and analysis of seminoma

a Partitioning the UMAP analysis in Fig 1b based on the donors of origin, and colors represent different donors (n=4 tumor samples).

b Bar plot showing the cell number of each cell type in four samples. Source data are provided as a Source data file.

c PCA illustrated the similarity of gene expression levels for each cell type across four samples. Different colors represent cell types, while shapes represent different samples. The cells circled in red are tumor cells.

d IF staining for OCT4/POU5F1 and SOX17 (seminoma tumor cells markers), CD19 (B cells marker) and CD68 (Macrophage marker). Scale bar, 50 μm.

e Additional markers expression pattern of cell types in Fig 1b**.**

f InferCNV plot of tumor cells.

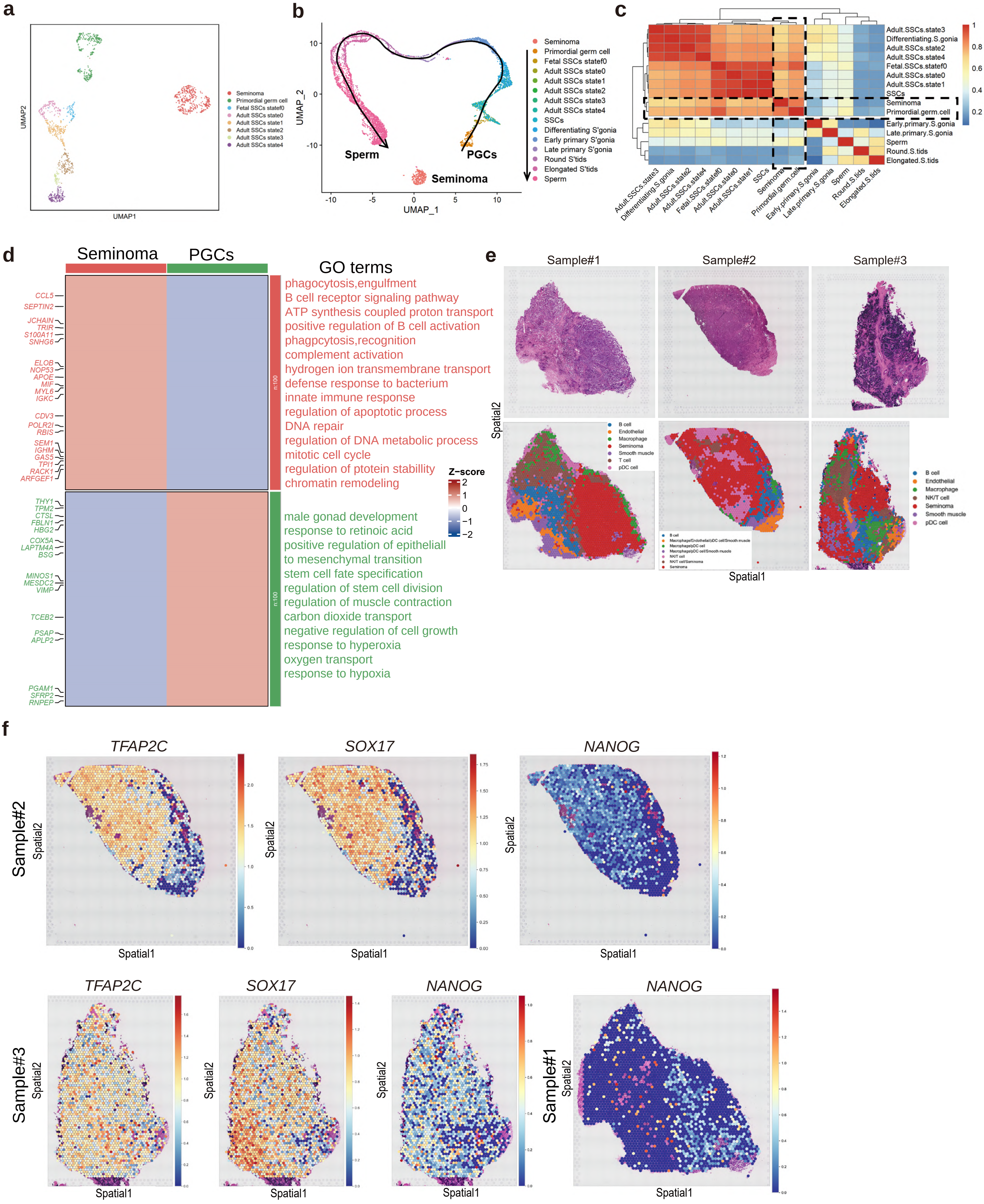

a
UMAP1
UMAP2
Seminoma
Primordial germ cell
Fetal SSCs state0
Adult SSCs state0
Adult SSCs state1
Adult SSCs state2
Adult SSCs state3
Adult SSCs state4
b
UMAP_1
UMAP_2
Sperm
PGCs
Seminoma
Seminoma
Primordial germ cell
Fetal SSCs statef0
Adult SSCs state0
Adult SSCs state1
Adult SSCs state2
Adult SSCs state3
Adult SSCs state4
SSCs
Differentiating S'gonia
Early primary S'gonia
Late primary S'gonia
Round S'tids
Elongated S'tids
Sperm
c
Adult.SSCs.state3
Differentiating.S.gonia
Adult.SSCs.state2
Adult.SSCs.state4
Fetal.SSCs.statef0
Adult.SSCs.state0
Adult.SSCs.state1
SSCs
Seminoma
Primordial.germ.cell
Early.primary.S.gonia
Late.primary.S.gonia
Sperm
Round.S.tids
Elongated.S.tids
d
Seminoma
PGCs
GO terms
CCL5
SEPTIN2
JCHAIN
TRIR
S100A11
SNHG6
ELOB
NOP53
APOE
MIF
MYL6
IGKC
CDV3
POLR2I
RBIS
SEM1
IGHM
GAS5
TPI1
RACK1
ARFGEF1
phagocytosis,engulfment
B cell receptor signaling pathway
ATP synthesis coupled proton transport
positive regulation of B cell activation
phagpcytosis,recognition
complement activation
hydrogen ion transmembrane transport
defense response to bacterium
innate immune response
regulation of apoptotic process
DNA repair
regulation of DNA metabolic process
mitotic cell cycle
regulation of ptotein stabillity
chromatin remodeling
Z-score
THY1
TPM2
CTSL
FBLN1
HBG2
COX5A
LAPTM4A
BSG
MINOS1
MESDC2
VIMP
TCEB2
PSAP
APLP2
PGAM1
SFRP2
RNPEP
male gonad development
response to retinoic acid
positive regulation of epitheliall
to mesenchymal transition
stem cell fate specification
regulation of stem cell division
regulation of muscle contraction
carbon dioxide transport
negative regulation of cell growth
response to hyperoxia
oxygen transport
response to hypoxia
e
Sample#1
Sample#2
Sample#3
Spatial2
Spatial1
B cell
Endothelial
Macrophage
Seminoma
Smooth muscle
T cell
pDC cell
NK/T cell
f
TFAP2C
SOX17
NANOG
Sample#2
Sample#3
Sample#1
Spatial1
Spatial2

Supplementary Figure 2. Comparisons of seminoma and different germ cell stages

a UMAP plot of seminoma (n=4 tumor samples) and early germ cells (n=9 samples)(n= 1320 cells). Details refer to Data availability statement.

b UMAP plot of seminoma (n=4 tumor samples) and male germ cells (n=12 germ cell samples) (n= 6108 cells). Curve with arrow represents male germ cell development.

c Correlation analysis among cell types shown in (Supplementary Fig. 2b) was calculated using Pearson's correlation coefficients. The black dotted box highlights the part with highest similarity between seminoma and PGCs.

d GO term enrichment of genes specifically expressed in seminoma tumor cells or PGCs. Source data are provided as a Source data file.

e Hematoxylin and eosin (H&E) staining (upper) of tissue sections and cell types annotation (below) of spatial transcriptome spots in three seminoma samples.

f Expression patterns of *TFAP2C*, *SOX17* and *NANOG* in two additional tumor samples used for spatial transcriptomic profiling.

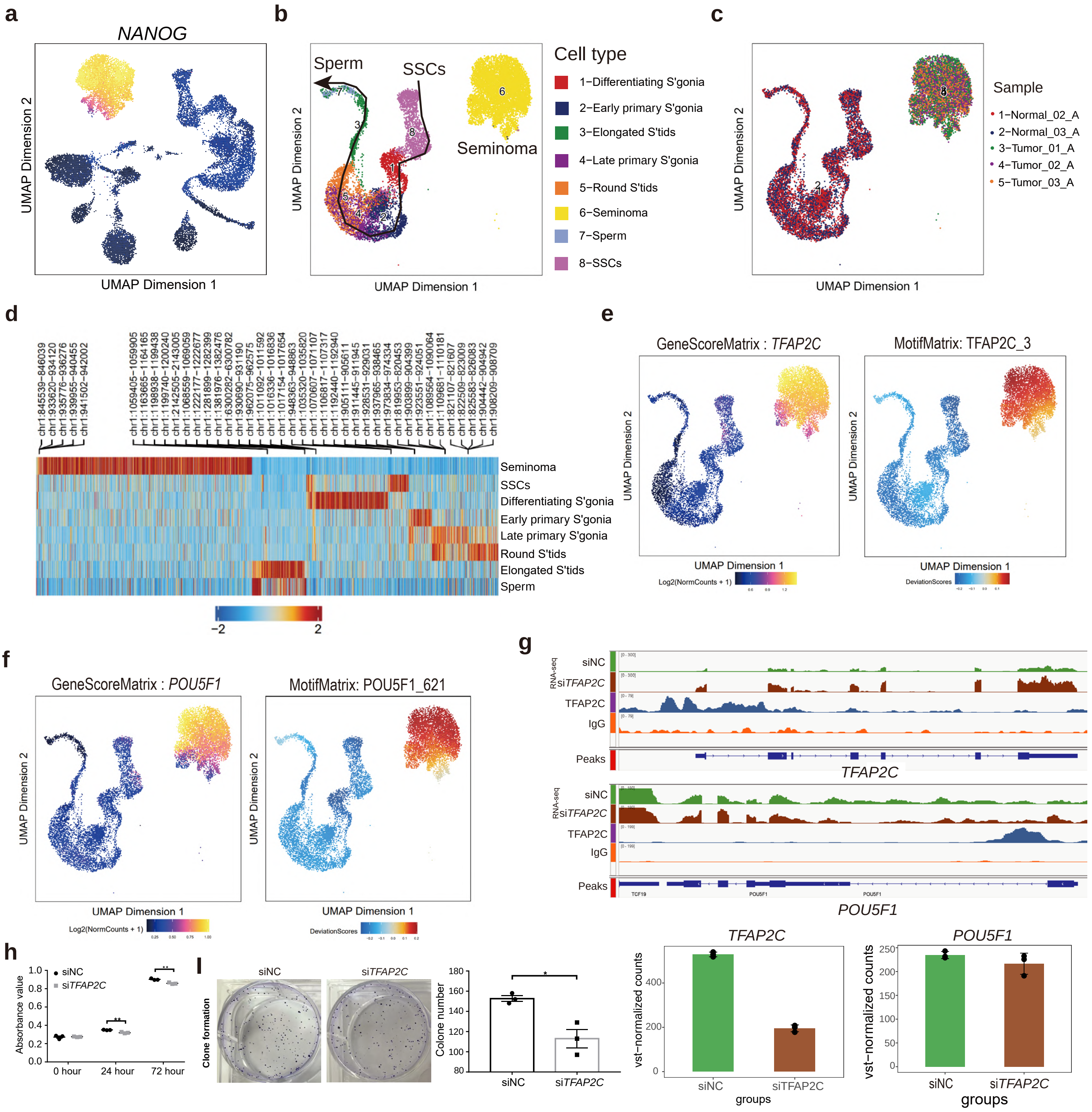
a
NANOG
UMAP Dimension 2
UMAP Dimension 1
b
Sperm
SSCs
Seminoma
UMAP Dimension 2
UMAP Dimension 1
Cell type
1−Differentiating S'gonia
2−Early primary S'gonia
3−Elongated S'tids
4−Late primary S'gonia
5−Round S'tids
6−Seminoma
7−Sperm
8−SSCs
c
UMAP Dimension 2
UMAP Dimension 1
Sample
1−Normal_02_A
2−Normal_03_A
3−Tumor_01_A
4−Tumor_02_A
5−Tumor_03_A
d
chr1:845539−846039
chr1:933620−934120
chr1:935776−936276
chr1:939955−940455
chr1:941502−942002
chr1:1059405−1059905
chr1:1163665−1164165
chr1:1198938−1199438
chr1:1199740−1200240
chr1:2142505−2143005
chr1:1068559−1069059
chr1:1222177−1222677
chr1:1281899−1282399
chr1:1381976−1382476
chr1:6300282−6300782
chr1:930690−931190
chr1:962075−962575
chr1:1011092−1011592
chr1:1016336−1016836
chr1:1017154−1017654
chr1:948363−948863
chr1:1035320−1035820
chr1:1070607−1071107
chr1:1106817−1107317
chr1:1192440−1192940
chr1:905111−905611
chr1:911445−911945
chr1:928531−929031
chr1:937965−938465
chr1:973834−974334
chr1:819953−820453
chr1:903899−904399
chr1:923551−924051
chr1:1089564−1090064
chr1:1109681−1110181
chr1:821107−821607
chr1:822509−823009
chr1:825583−826083
chr1:904442−904942
chr1:908209−908709
Seminoma
SSCs
Differentiating S'gonia
Early primary S'gonia
Late primary S'gonia
Round S'tids
Elongated S'tids
Sperm
−2
2
e
GeneScoreMatrix : TFAP2C
MotifMatrix: TFAP2C_3
UMAP Dimension 2
UMAP Dimension 1
Log2(NormCounts + 1)
DeviationScores
f
GeneScoreMatrix : POU5F1
MotifMatrix: POU5F1_621
UMAP Dimension 2
UMAP Dimension 1
Log2(NormCounts + 1)
DeviationScores
g
RNA-seq
siNC
siTFAP2C
TFAP2C
IgG
Peaks
TFAP2C
TCF19
POU5F1
POU5F1
h
siNC
siTFAP2C
Absorbance value
1.0
0.8
0.6
0.4
0.2
0.0
0 hour
24 hour
72 hour
**
**
i
siNC
siTFAP2C
Clone formation
Colone number
180
160
140
120
100
80
*
siNC
siTFAP2C
TFAP2C
vst−normalized counts
400
200
0
siNC
siTFAP2C
groups
POU5F1
vst−normalized counts
250
200
150
100
50
0
siNC
siTFAP2C
groups

Supplementary Figure 3. Key transcription factors expression in the scATAC-seq data
a Expression pattern (gene score) of seminoma marker *SOX17* in scATAC-seq data.
b UMAP plot of seminoma and adult normal germ cells (n=14995 cells) subset from Fig 3b (n= 1 tumor sample, 3 independent replicates and 1 normal sample, 2 independent replicates), annotated with scRNA-seq data.
c Sample information of (Supplementary Figure. 3b).
d Heatmap showing cell type specific peaks.
e The levels of gene scores (promoter openness) and motifs enriched for *TFAP2C* in cell type show in (Supplementary Figure. 3b).
f The levels of gene scores (promoter openness) and motifs enriched for *POU5F1* in cell type show in (Supplementary Figure. 3b).
g Coverage plots of *TFAP2C* and *POU5F1* in siNC, si*TFAP2C* and CUT&Tag enriched. Bar plot showed the expression level of *TFAP2C* and *POU5F1* in RNA-seq data. Data are presented as mean values +/- SEM" as appropriate. n=3 independent experiments. Source data are provided as a Source data file.
h CCK8 absorbance in siNC and si*TFAP2C* group. P value was calculated by two-sided Wilcoxon rank-sum test. ***P* <0.05. Data are presented as mean values +/- SEM" as appropriate. n=3 independent experiments. The black dots represent the number of independent experiments of the siNC and the black squares represent the number of independent experiments of the si*TFAP2C*. Source data are provided as a Source data file.
i Cell clone formation in siNC and si*TFAP2C* group. P value was calculated by two-sided Wilcoxon rank-sum test. **P* <0.05. Data are presented as mean values +/- SEM" as appropriate. n=3 independent experiments. The black dots represent the number of independent experiments of the siNC and the black squares represent the number of independent experiments of the si*TFAP2C*. Source data are provided as a Source data file.

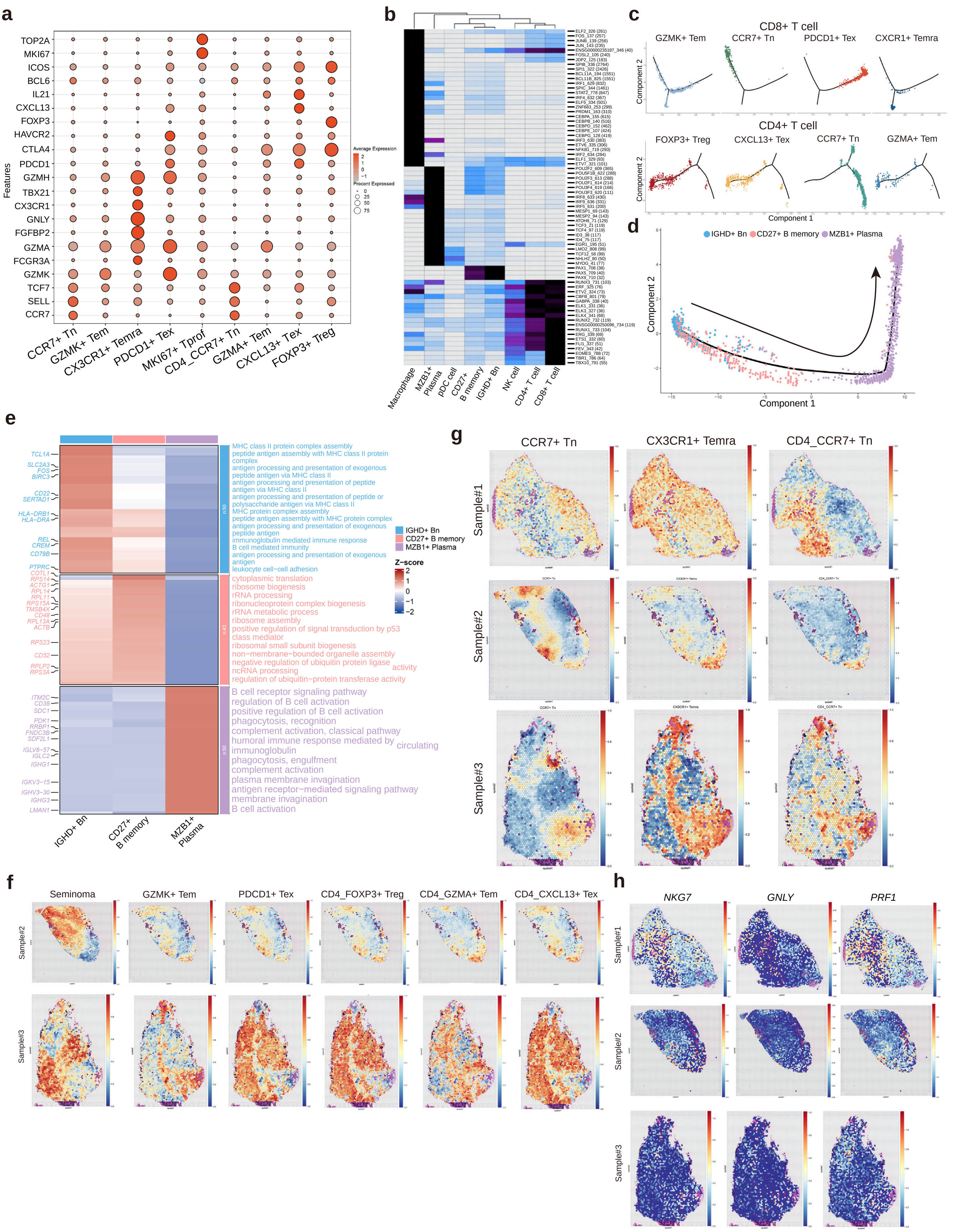
a
TOP2A
MKI67
ICOS
BCL6
IL21
CXCL13
FOXP3
HAVCR2
CTLA4
PDCD1
GZMH
TBX21
CX3CR1
GNLY
FGFBP2
GZMA
FCGR3A
GZMK
TCF7
SELL
CCR7
Features
Average Expression
Percent Expressed
CCR7+ Tn
GZMK+ Tem
CX3CR1+ Temra
PDCD1+ Tex
MKI67+ Tprof
CD4_CCR7+ Tn
GZMA+ Tem
CXCL13+ Tex
FOXP3+ Treg
b
Macrophage
MZB1+ Plasma
pDC cell
CD27+ B memory
IGHD+ Bn
NK cell
CD4+ T cell
CD8+ T cell
c
CD8+ T cell
GZMK+ Tem
CCR7+ Tn
PDCD1+ Tex
CXCR1+ Temra
Component 2
CD4+ T cell
FOXP3+ Treg
CXCL13+ Tex
CCR7+ Tn
GZMA+ Tem
Component 1
d
IGHD+ Bn
CD27+ B memory
MZB1+ Plasma
Component 2
Component 1
e
TCL1A
SLC2A3
FOS
BIRC3
CD22
SERTAD1
HLA−DRB1
HLA−DRA
REL
CREM
CD79B
PTPRC
COTL1
RPS14
ACTG1
RPL14
RPL11
RPS15A
TMSB4X
CD48
RPL13A
ACTB
RPS23
CD52
RPLP2
RPS3A
ITM2C
CD38
SDC1
PDK1
RRBP1
FNDC3B
SDF2L1
IGLV6−57
IGLC2
IGHG1
IGKV3−15
IGHV3−30
IGHG3
LMAN1
IGHD+ Bn
CD27+ B memory
MZB1+ Plasma
MHC class II protein complex assembly
peptide antigen assembly with MHC class II protein complex
antigen processing and presentation of exogenous peptide antigen via MHC class II
antigen processing and presentation of peptide antigen via MHC class II
antigen processing and presentation of peptide or polysaccharide antigen via MHC class II
MHC protein complex assembly
peptide antigen assembly with MHC protein complex
antigen processing and presentation of exogenous peptide antigen
immunoglobulin mediated immune response
B cell mediated immunity
antigen processing and presentation of exogenous antigen
leukocyte cell−cell adhesion
cytoplasmic translation
ribosome biogenesis
rRNA processing
ribonucleoprotein complex biogenesis
rRNA metabolic process
ribosome assembly
positive regulation of signal transduction by p53 class mediator
ribosomal small subunit biogenesis
non−membrane−bounded organelle assembly
negative regulation of ubiquitin protein ligase activity
ncRNA processing
regulation of ubiquitin−protein transferase activity
B cell receptor signaling pathway
regulation of B cell activation
positive regulation of B cell activation
phagocytosis, recognition
complement activation, classical pathway
humoral immune response mediated by circulating immunoglobulin
phagocytosis, engulfment
complement activation
plasma membrane invagination
antigen receptor−mediated signaling pathway
membrane invagination
B cell activation
IGHD+ Bn
CD27+ B memory
MZB1+ Plasma
Z−score
g
CCR7+ Tn
CX3CR1+ Temra
CD4_CCR7+ Tn
Sample#1
Sample#2
Sample#3
f
Seminoma
GZMK+ Tem
PDCD1+ Tex
CD4_FOXP3+ Treg
CD4_GZMA+ Tem
CD4_CXCL13+ Tex
Sample#2
Sample#3
h
NKG7
GNLY
PRF1
Sample#1
Sample#2
Sample#3

Supplementary Figure 4. Immune cell subtypes

a Marker genes expression of immune cell subtypes show in Fig 4a.

b TF binding motifs enriched in immune cell types.

c Development trajectory of CD8+ T cells and CD4+ T cells subtypes.

d Developmental trajectory of B cell subtypes. The smoothed line and arrow represent the visualization of the trajectory across different subtypes (naive, memory and plasma).

e GO terms enriched in B cell subtypes.

f Expression position of seminoma tumor cells and T cell subtypes in spatial transcriptome data of seminoma sample #2 and sample #3. These subtypes consistent with Fig 4e (expressed in sample #1).

g Spatial transcriptome expression position of T cell subtypes in three seminoma samples.

h Expression of immune cell cytotoxic factors in three seminoma spatial transcriptome samples.

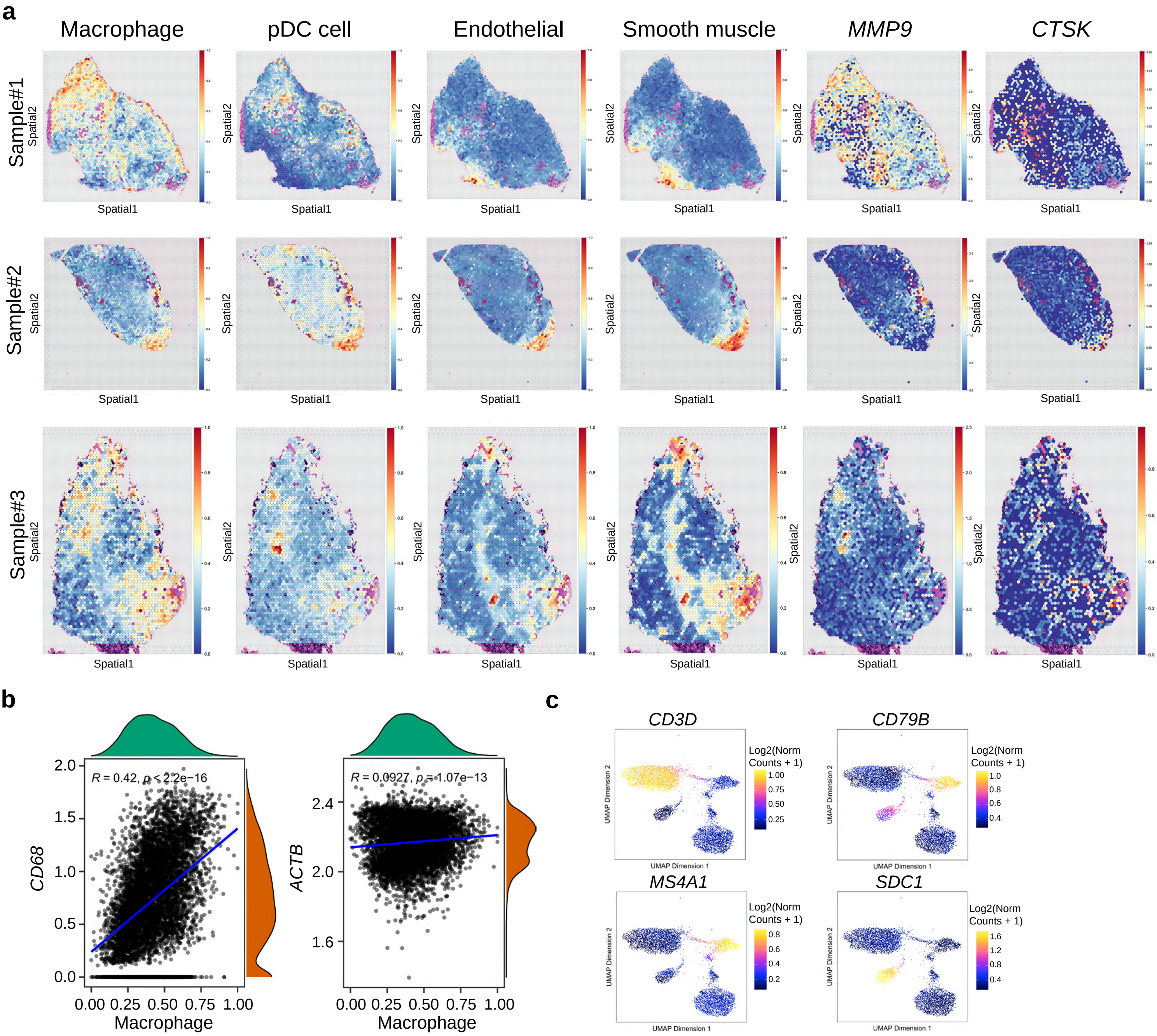

Supplementary Figure 5. Spatial expression pattern of macrophage and *MMP9*, *CTSK*

a Spatial transcriptome expression patterns of *MMP9*, *CTSK* and selected cell types from scRNA-seq data in three seminoma samples.

b Correlation analysis of *CD68* and *ACTB* expression with macrophage location in spatial transcriptome data using Pearson's correlation coefficients, as positive control and negative control for Fig 5d, respectively. Points represent the cells in macrophage. Exact pvalue of *CD68* and macrophage: 6.76E-272; Exact pvalue of *ACTB* and macrophage: 1.07E-13. Source data are provided as a Source data file.

c Other marker genes expression in immune cell scATAC-seq data. *CD3D*: T cells marker. *CD79B* and *MS4A1*: B cells markers. *SDC1*: plasma cells markers.

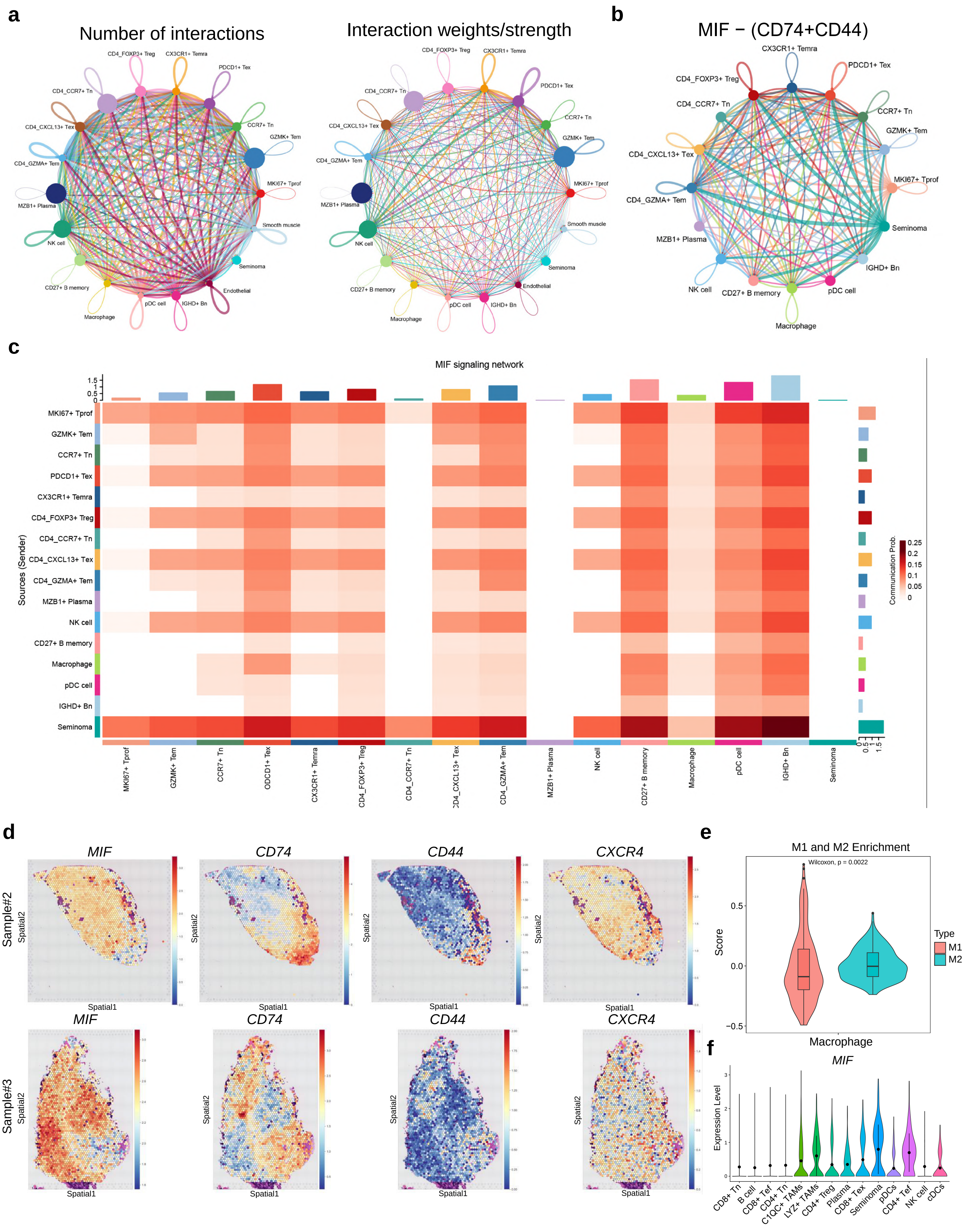

a
Number of interactions
Interaction weights/strength
CD4_FOXP3+ Treg
CX3CR1+ Temra
PDCD1+ Tex
CD4_CCR7+ Tn
CCR7+ Tn
CD4_CXCL13+ Tex
GZMK+ Tem
CD4_GZMA+ Tem
MKI67+ Tprof
MZB1+ Plasma
Smooth muscle
NK cell
Seminoma
CD27+ B memory
Endothelial
pDC cell
IGHD+ Bn
Macrophage
b
MIF − (CD74+CD44)
c
MIF signaling network
Sources (Sender)
Communication Prob.
d
MIF
CD74
CD44
CXCR4
Sample#2
Sample#3
Spatial1
Spatial2
e
M1 and M2 Enrichment
Wilcoxon, p = 0.0022
Score
Type
M1
M2
Macrophage
f
MIF
Expression Level
CD8+ Tn
B cell
CD8+ Tef
CD4+ Tn
C1QC+ TAMs
LYZ+ TAMs
CD4+ Treg
Plasma
CD8+ Tex
Seminoma
pDCs
CD4+ Tef
NK cell
cDCs

Supplementary Figure 6. Cell communications between tumor cell and immune cell
a The number and weight of interactions between all cells in the microenvironment. **b** The interactions of all cells through MIF-(CD74+CD44).
c Heatmap showing communication probability of MIF pathway in each pair of cell types. Variables on x axis or y axis represent secreting cells or target cells, respectively. **d** The ligand (*MIF*) and receptor (*CD74*, *CD44* and *CXCR4*) of MIF pathway expression pattern in the other two spatial transcriptomes samples.
e Violin plot showing the enrichment of M1 and M2 signature in seminoma macrophage (n=4 samples). P value was calculated by two-sided Wilcoxon rank-sum test. Box plots show median (center line), the upper and lower quantiles (box), and the range of the data (whiskers). The genes associated M1 and M2 in Supplementary Data 2. Source data are provided as a Source data file.
f Violin plot showing the expression of *MIF* in seminoma which metastasized to the lymph nodes (n= 6805 cells). The black points show the mean.

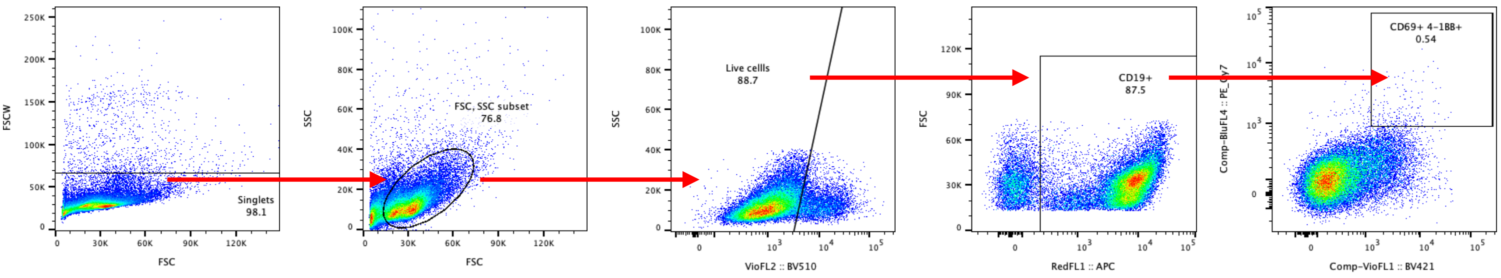

Gating strategy and a representative dot plot for identification of the percentage of activated CD19$^+$ B cells. Single-cell suspensions were isolated from B cells by MojoSort™ Human Pan B Cell Isolation Kit from healthy controls. We utilized a single-cell gating strategy for flow cytometric analysis. Firstly, forward scatter (FSC) and side scatter (SSC) were used to exclude doublets to obtain the 76.8% of pure single cells, then the Zombie Aqua fixable viability kit (Biolegend, channel: BV510) was used to distinguish between live (88.7%) and dead cells. Finally, CD19$^+$ (Biolegend, APC) B cells and CD69$^+$ (BD, BV421) 4-1BB$^+$ (Biolegend, PE-Cy-7) activated B cells were analyzed with antibodies. The representative dot plots of CD69$^+$4-1BB$^+$ activated B cells are shown in Figure 6F in the manuscript.
