# Supplementary material for: Single-cell multi-omics analysis of human testicular germ cell tumor reveals its molecular features and microenvironment
Source: Nat Commun. 2023 Dec 20;14:8462. doi: 10.1038/s41467-023-44305-9 (PMC10733385; doi:10.1038/s41467-023-44305-9)
Supplement: Supplementary file 3 — Description of Additional Supplementary Files [file 41467_2023_44305_MOESM3_ESM.pdf]

Description of Additional Supplementary Files

Title: Supplementary Data 1
Description: Clinical characteristics of seminoma patients.

Title: Supplementary Data 2
Description: Gene signatures related to Degradation of the extracellular matrix pathway, macrophage M1 and M2.

Title: Supplementary Data 3
Description: Cell distribution of early germ cells and seminoma in each PC in Figure 2c.
