# Supplementary material for: Single-cell multi-omics analysis of human testicular germ cell tumor reveals its molecular features and microenvironment
Source: Nat Commun. 2023 Dec 20;14:8462. doi: 10.1038/s41467-023-44305-9 (PMC10733385; doi:10.1038/s41467-023-44305-9)
Supplement: Supplementary file 5 — Reporting Summary [file 41467_2023_44305_MOESM5_ESM.pdf]

nature portfolio

Corresponding author(s): Jingtao Guo, Hao Bo, Zhe Zhang

Last updated by author(s): Dec 6,2023

Reporting Summary

Nature Portfolio wishes to improve the reproducibility of the work that we publish. This form provides structure for consistency and transparency in reporting. For further information on Nature Portfolio policies, see our Editorial Policies and the Editorial Policy Checklist.

Statistics

For all statistical analyses, confirm that the following items are present in the figure legend, table legend, main text, or Methods section.

n/a	Confirmed	
☒	☐	The exact sample size (*n*) for each experimental group/condition, given as a discrete number and unit of measurement
☐	☒	A statement on whether measurements were taken from distinct samples or whether the same sample was measured repeatedly
☐	☒	The statistical test(s) used AND whether they are one- or two-sided<br>*Only common tests should be described solely by name; describe more complex techniques in the Methods section.*
☐	☒	A description of all covariates tested
☐	☒	A description of any assumptions or corrections, such as tests of normality and adjustment for multiple comparisons
☐	☒	A full description of the statistical parameters including central tendency (e.g. means) or other basic estimates (e.g. regression coefficient) AND variation (e.g. standard deviation) or associated estimates of uncertainty (e.g. confidence intervals)
☐	☒	For null hypothesis testing, the test statistic (e.g. *F*, *t*, *r*) with confidence intervals, effect sizes, degrees of freedom and *P* value noted<br>*Give P values as exact values whenever suitable.*
☒	☐	For Bayesian analysis, information on the choice of priors and Markov chain Monte Carlo settings
☐	☒	For hierarchical and complex designs, identification of the appropriate level for tests and full reporting of outcomes
☐	☒	Estimates of effect sizes (e.g. Cohen's *d*, Pearson's *r*), indicating how they were calculated

Our web collection on statistics for biologists contains articles on many of the points above.

Software and code

Policy information about availability of computer code

Data collection	scRNA-seq data were collected by Chromium single cell controller(10x Genomics)with built-in software. Spatial transcriptome sequencing data were collected by Visium CytAssist (10x Genomics) with built-in software. scATAC-seq data obtained from the DIPSEQ T1 sequencer platform (BGI).
Data analysis	All scRNA-seq data were processed with CellRanger v7.0.0, R v4.2.1, Rstudio v2022.12.0-353 and the following R package were used: BiocManager v1.30.19, devtools v2.4.5, RColorBrewer v1.1-3, Matrix v1.5-3, pheatmap v1.0.12, patchwork v1.1.1, ggplot2 v3.4.1, dplyr v1.0.9, tidyselect v1.2.0, Seurat v4.1.1, scRNAtoolVis v0.0.4, jjAnno v0.0.3, Cellchat v1.4.0, Monocle v2.26.0, inferCNV V1.12.0.<br>DEseq2 v1.36.0 and ggvolcano v0.0.2Ewere used for bulk and scRNA-seq analysis.<br>All Spatial transcriptome data were processed Spaceranger v1.0.0, python v3.9.12, PyCharm v2022.1.3 and the following python package were used: anndata v0.8.0, scanpy v1.9.1, pandas v1.5.3, numpy v1.21.6, matplotlib v3.5.1, scanorama v1.7.2.<br>ScATAC-seq data were processed with PISA v0.12 and analyzed using R package ArchR v1.0.2 and Cairo v1.6.0. MACS2 v2.2.7.1 was used for peaks calling.<br>CUT&Tag-seq data were analyzed with FastQC v0.11.9, trim_galore v0.6.7, bowtie2 v2.2.5, samtools v.1.6, bamCoverage v3.5.1, deeptools v3.5.1, MACS2 v2.2.7.1, homer v23.02.2, chipseeker v1.32.1.

For manuscripts utilizing custom algorithms or software that are central to the research but not yet described in published literature, software must be made available to editors and reviewers. We strongly encourage code deposition in a community repository (e.g. GitHub). See the Nature Portfolio guidelines for submitting code & software for further information.

Data

Policy information about availability of data

All manuscripts must include a data availability statement. This statement should provide the following information, where applicable:

- Accession codes, unique identifiers, or web links for publicly available datasets
- A description of any restrictions on data availability
- For clinical datasets or third party data, please ensure that the statement adheres to our policy

The raw sequence data generated in this study have been deposited in the Genome Sequence Archive at National Genomics Data Center, China National Center for Bioinformation/Beijing Institute of Genomics, Chinese Academy of Sciences (https://ngdc.cncb.ac.cn/gsa-human, accession no. HRA004404 for scRNA-seq data (https://bigd.big.ac.cn/gsa-human/browse/HRA004404); accession no. HRA004502 for scATAC-seq data (https://bigd.big.ac.cn/gsa-human/browse/HRA004502); accession no. HRA004398 for 10X Visium spatial transcriptome data (https://bigd.big.ac.cn/gsa-human/browse/HRA004398) and accession no. HRA006112 for CUT&Tag data (https://bigd.big.ac.cn/gsa-human/browse/HRA006112)) that are publicly accessible. The processed expression matrices in this paper have been deposited in the OMIX, China National Center for Bioinformation / Beijing Institute of Genomics, Chinese Academy of Sciences (https://ngdc.cncb.ac.cn/omix, accession no. OMIX004217). The publicly available data of normal germ cell scRNA-seq and infant data included in early germ cells used in this study are available in the GEO database under accession code GSE120508 [https://www.ncbi.nlm.nih.gov/geo/query/acc.cgi?acc=GSE120508]. The publicly available data of early germ cell scRNA-seq used in this study are available in the GEO database under accession code GSE143356 [https://www.ncbi.nlm.nih.gov/geo/query/acc.cgi?acc=GSE143356].
The remaining data are available within the Article, Supplementary Information, Source Data file or from the corresponding author upon request. Source data are provided with this paper. This study did not generate any unique code or algorithm.

Research involving human participants, their data, or biological material

Policy information about studies with human participants or human data. See also policy information about sex, gender (identity/presentation), and sexual orientation and race, ethnicity and racism.

Reporting on sex and gender	All of samples included in our study are male(biological attribute). Because seminoma is a type of testicular germ cell tumor.
Reporting on race, ethnicity, or other socially relevant groupings	The samples used for single-cell sequencing were obtained from Caucasian males, while the remaining samples were obtained from Asian males.
Population characteristics	We collected samples that were in a naive treatment state. The age of four samples for single-cell RNA sequencing are 28 ,34,63,38 years old, respectively. The age of three patients for single-cell ATAC-seq are 33,55,31 years old, and two samples for spatial transcriptome sequencing are 34, 35 years old, respectively.
Recruitment	Samples were obtained from partner hospitals.
Ethics oversight	Seminoma samples used for scRNA-seq were obtained from four individuals, with written consent, through the University of Utah Andrology laboratory (IRB approved protocol #00075836). Studies with human specimens used for scATAC-seq and 10X Visium profiling were approved by the Ethics Committee of the Affiliated Cancer Hospital of Xiangya School of Medicine, Central South University (2021KYKS-46).

Note that full information on the approval of the study protocol must also be provided in the manuscript.

Field-specific reporting

Please select the one below that is the best fit for your research. If you are not sure, read the appropriate sections before making your selection.

[x] Life sciences [ ] Behavioural & social sciences [ ] Ecological, evolutionary & environmental sciences

For a reference copy of the document with all sections, see nature.com/documents/nr-reporting-summary-flat.pdf

Life sciences study design

All studies must disclose on these points even when the disclosure is negative.

Sample size	No statistical methods were used to predetermine sample size of scRNA-seq, scATAC-seq libraries. We collected 4 patients from the University of Utah Health Sciences Center for scRNA-seq. All samples that passed the single-cell library QC were included in the study. We collected 3 seminoma patient samples for spatial transcriptome sequencing from The Affiliated Cancer Hospital of Xiangya School of Medicine of Central South University. scATAC-seq used 1 patient sample and 1 healthy control sample, both samples were from The Affiliated Cancer Hospital of Xiangya School of Medicine of Central South University. We also collected 6 individual seminoma patients for IHC/IF staining. All of these patient samples were primary seminoma that had not metastasized.
Data exclusions	All criteria for data exclusion were pre-established. Cells with less than 500 UMI counts, less than 200 genes, or greater than 20% of mitochondrial RNA counts were filtered.
Replication	For scRNA-seq analysis and spatial transcriptome sequencing, there is no replication for the human tumor samples. For scATAC-seq analysis, We performed three technical replications on individual samples. For scRNA-seq analysis and spatial transcriptome sequencing, there is no

replication for the human tumor samples. For scATAC-seq analysis, We performed three technical replications on individual samples.
For scRNA-seq analysis and spatial transcriptome sequencing, there is no replication for the human tumor samples. For scATAC-seq analysis, We performed three technical replications on individual samples.

Randomization: No randomization was performed for the human tumor samples because this is an observational study.

Blinding: Blinding was not considered appropriate for this study because this is an observational study. Our analyses and results were based on the cancer type of samples determined by pathologists.

Reporting for specific materials, systems and methods

We require information from authors about some types of materials, experimental systems and methods used in many studies. Here, indicate whether each material, system or method listed is relevant to your study. If you are not sure if a list item applies to your research, read the appropriate section before selecting a response.

Materials & experimental systems

n/a	Involved in the study	
☐	☒	Antibodies
☐	☒	Eukaryotic cell lines
☒	☐	Palaeontology and archaeology
☒	☐	Animals and other organisms
☒	☐	Clinical data
☒	☐	Dual use research of concern
☒	☐	Plants

Methods

n/a	Involved in the study	
☒	☐	ChIP-seq
☐	☒	Flow cytometry
☒	☐	MRI-based neuroimaging

Antibodies

Antibodies used:

Rabbit polyclonal anti-CD45 Dilution:1:400 Proteintech Cat# 20103-1-AP
Rabbit monoclonal anti-VIM Dilution:1:300 Cell Signaling Technology Cat# 5741T
Rabbit monoclonal anti-NANOG Dilution:1:100 Abcam Cat# ab109250
Mouse monoclonal anti-OCT4 Dilution:1:200 Santa Cat# sc-5279
Anti-CD3 Dilution:1:200 MXB Biotechnologies Cat# MAB-0740
Anti-PDCD1 Dilution:1:200 ProMab Biotechnologies Cat# P04417
Goat polyclonal anti-SOX17 Dilution:1:50 R&D systems Cat# AF1924
Rabbit monoclonal anti-CD19 Dilution:1:200 Abcam Cat# ab134114
Mouse monoclonal anti-CD68 Dilution:1:3200 Proteintech Cat# 66231-2-Ig
Rabbit polyclonal anti-MMP9 Dilution:1:400 Proteintech Cat# 10375-2-AP
Rabbit polyclonal anti-CTSK Dilution:1:200 Proteintech Cat# 11239-1-AP
Rabbit polyclonal anti-MIF Dilution:1:800 Proteintech Cat# 20415-1-AP
Mouse monoclonal anti-CD74 Dilution:1:1600 Proteintech Cat# 66390-1-Ig
Rabbit polyclonal anti-TFAP2C Dilution:1:100 Proteintech Cat No:14572-1-AP

Validation: All antibodies used in this study are commercially available. They are validated by the vendors for the specific assay and species used, with the validation reports available on the vendor's website.

Eukaryotic cell lines

Policy information about cell lines and Sex and Gender in Research

Cell line source(s): TCam-2 cell line was kindly gifted from Dr. Riko Kitazawa (Department of Diagnostic Pathology, Ehime University Hospital, Matsuyama, Japan)

Authentication: The cell line we used was not authenticated.

Mycoplasma contamination: We confirm the cell line tested negative for mycoplasma contamination.

Commonly misidentified lines (See ICLAC register): This is not applicable to out study.

Flow Cytometry

Plots

Confirm that:

- [x] The axis labels state the marker and fluorochrome used (e.g. CD4-FITC).
- [x] The axis scales are clearly visible. Include numbers along axes only for bottom left plot of group (a 'group' is an analysis of identical markers).
- [x] All plots are contour plots with outliers or pseudocolor plots.
- [x] A numerical value for number of cells or percentage (with statistics) is provided.

Methodology

Sample preparation	B cells examined in this study were derived from peripheral blood mononuclear cells (PBMCs) purified from peripheral blood by density gradient centrifugation with Lymphoprep (STEMCELL Technologies). Total CD19+ B cells were prepared with MojoSort™ Human Pan B Cell Isolation Kit (Catalog:480082, Biolegend). B cells were cultured in full RPMI 1640 medium in the presence of 10 ng/mL recombinant human MIF protein (Catalog: 300-69-5, peprotech) for 48h. In the last 24h, the culture medium was supplemented with 100 ng/mL LPS (Sigma-Aldrich) as previously described72-74.<br>Cell surface molecule staining was analyzed as previously reported using the flow cytometer (Cytec, USA) 72,75. Zombie Aqua fixable viability kit (catalog: 423101), APC-anti-human CD19 (catalog: 302212), PE-Cy7-anti-human 4-1BB (catalog: 309817) were purchased from Biolegend (USA). Brilliant Violet 421-anti-human CD69 (catalog: 562884) was purchased from BD(USA).
Instrument	Cytec, USA
Software	FlowJo 10.0
Cell population abundance	Up to 10^6 single viable cells were analyzed from cultured B cells.
Gating strategy	Information available on Fig 6F, and Methods sections. Live cells were firstly gated by FSC-A and SSC-A to exclude the debris, followed by FSC-H to gate single cells. Dead cells were exclued by using viability dye. CD69+&4-1BB+ cells were used to identify activated B cells.

- [ ] Tick this box to confirm that a figure exemplifying the gating strategy is provided in the Supplementary Information.
